# Beyond Reflux Grade: Long-Term Outcomes of Children with Grade 1 Vesicoureteral Reflux

**DOI:** 10.3390/children13081036

**Published:** 2026-08-03

**Authors:** Hasan Deliağa, Halil Tosun, Bilge Karabulut, Hüseyin Tuğrul Tiryaki

**Affiliations:** 1 Department of Pediatric Urology, Bursa Medical Faculty, University of Health Sciences, 16310 Bursa, Türkiye; 2 Department of Pediatric Urology, Faculty of Medicine, Erciyes University, 38280 Kayseri, Türkiye; tosunhalil38@gmail.com; 3 Department of Pediatric Urology, Ankara Bilkent City Hospital, University of Health Sciences, 06800 Ankara, Türkiye; bilgekarabulut@hotmail.com (B.K.); httiryaki@hotmail.com (H.T.T.)

**Keywords:** vesicoureteral reflux, grade 1 VUR, DMSA scintigraphy, renal scarring, urinary tract infection, pediatric nephrology

## Abstract

**Highlights:**

**What are the main findings?**

- Children with Grade 1 VUR demonstrated heterogeneous clinical phenotypes within a tertiary referral population.
- Older age at diagnosis and abnormal baseline DMSA findings were associated with renal adverse outcomes during long-term follow-up.

**What are the implications of the main findings?**

- Clinical interpretation of Grade 1 VUR should consider the broader clinical context rather than the reflux grade alone.
- These findings are descriptive and hypothesis-generating and warrant validation in prospective comparative studies.

**Abstract:**

**Background:** Grade 1 vesicoureteral reflux (VUR) is generally considered a low-risk condition and is commonly grouped with other low-grade reflux categories in the literature. Consequently, long-term outcome data specifically addressing Grade 1 VUR remain limited. We evaluated the long-term renal and infectious outcomes of children with Grade 1 VUR and characterized their principal associated clinical phenotypes. **Methods:** This retrospective cohort study included children diagnosed with Grade 1 VUR between 2008 and 2025 at a tertiary pediatric urology center. Primary outcomes were breakthrough urinary tract infection (UTI), proteinuria, and new renal scar formation. Principal associated clinical phenotypes and factors associated with adverse outcomes were evaluated using univariable analyses. **Results:** A total of 132 children with Grade 1 VUR involving 154 refluxing renal units were included. Median age at diagnosis was 6 years (IQR 4–9.25), and median follow-up was 8 years (IQR 5–9). A principal associated clinical phenotype was identified in all patients, most commonly voiding dysfunction (38.6%), recurrent UTI (28.0%), and renal anomalies (25.0%). Breakthrough UTI occurred in 46 patients (34.8%), proteinuria in 6 (4.5%), and new renal scar formation was identified in 7 of the 70 patients who underwent paired baseline and follow-up DMSA examinations performed according to clinical indications (10.0%). Renal adverse outcomes (proteinuria and/or new renal scar formation) were identified in 10 patients. Incident renal scarring was assessable only in the selectively imaged paired DMSA subgroup. Older age at diagnosis (*p* < 0.001) and abnormal baseline DMSA findings (80.0% vs. 41.8%, *p* = 0.040) were associated with renal adverse outcomes, while female sex was associated with breakthrough UTI (*p* = 0.009). **Conclusions:** In this tertiary referral cohort of children with Grade 1 VUR and associated clinical conditions, heterogeneous clinical outcomes were observed during long-term follow-up. These findings highlight the importance of interpreting Grade 1 VUR within the broader clinical context of the child; however, the independent contribution of reflux itself cannot be determined from the present study. These findings should therefore be regarded as descriptive and hypothesis-generating.

## 1. Introduction

Vesicoureteral reflux (VUR) is a common pediatric urinary tract condition and remains clinically important because of its association with urinary tract infection (UTI), pyelonephritis, renal scarring, hypertension, proteinuria, and long-term renal morbidity [1,2]. Although the clinical relevance of VUR has traditionally been interpreted according to reflux grade, the relationship between anatomical reflux severity and renal outcome is not always straightforward [1,2]. Patient-specific factors, including recurrent infection, bladder–bowel dysfunction, voiding dysfunction, congenital renal or urinary tract anomalies, and baseline renal parenchymal abnormalities, may substantially modify clinical risk [1,2,3].

Grade 1 VUR is usually regarded as the mildest form of reflux because retrograde flow is limited to a non-dilated ureter without involvement of a dilated collecting system [4]. Consequently, it is often assumed to have a benign clinical course and may receive less attention than dilating or high-grade reflux [1]. This perception is partly understandable, as high-grade reflux is more clearly associated with renal injury and has formed the basis of many historical treatment strategies [1,5]. However, the assumption that Grade 1 VUR is clinically negligible may be overly simplistic [6].

Importantly, Grade 1 VUR has not been adequately characterized as an isolated clinical subgroup in the major VUR literature. Classic randomized surgical versus medical management trials predominantly focused on moderate- and high-grade reflux, whereas more recent studies frequently grouped Grade 1 VUR with other low-grade categories [7,8,9,10,11,12,13,14]. In the RIVUR trial, children with Grades I–IV reflux were included, but Grade I reflux represented only 11% of the cohort, while Grades II and III reflux predominated [11]. Therefore, long-term outcome data specifically focused on Grade 1 VUR remain limited.

This evidence gap may have contributed to the underestimation of Grade 1 VUR in clinical practice. Anatomically, Grade 1 VUR is low grade; however, the patient carrying Grade 1 VUR may not necessarily be low risk. In some children, Grade 1 VUR may coexist with recurrent UTI, voiding dysfunction, renal anomalies, stone disease, familial Mediterranean fever, abnormal DMSA findings, or other conditions that determine renal prognosis more strongly than reflux grade itself [15,16]. In this context, Grade 1 VUR may function less as an isolated benign radiologic finding and more as a marker accompanying a broader urinary or systemic phenotype [2].

The aim of the present study was to evaluate the long-term renal and infectious outcomes of children with Grade 1 VUR and to describe the spectrum of principal associated clinical phenotypes in this group. We specifically assessed breakthrough UTI, proteinuria, and new renal scar formation during follow-up, with the hypothesis that Grade 1 VUR should not be dismissed solely on the basis of its low anatomical grade.

## 2. Materials and Methods

### 2.1. Study Design and Patient Population

**Figure 1:**
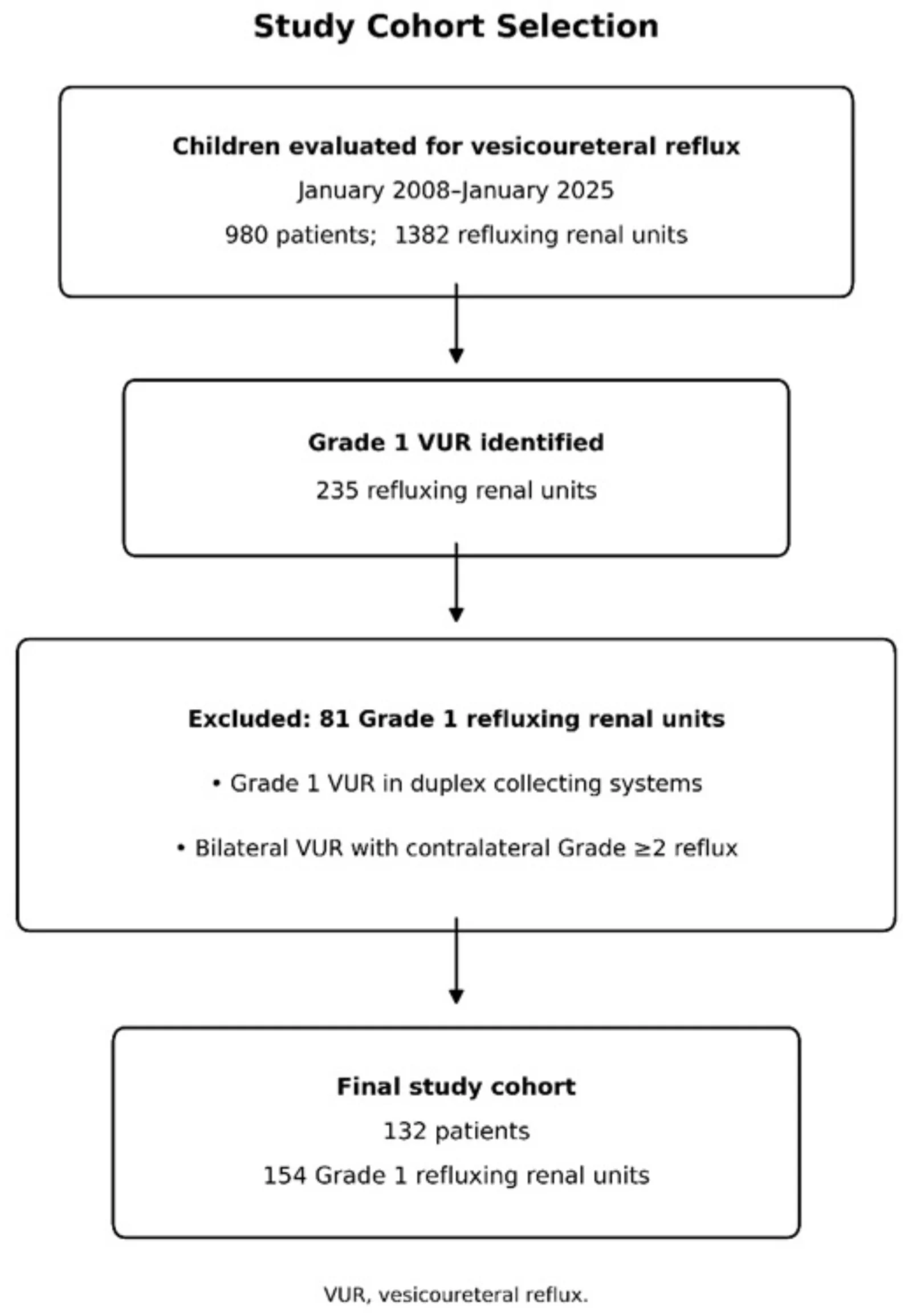
Flow diagram illustrating renal-unit-based cohort selection and derivation of the final patient cohort. During the study period (January 2008–January 2025), 980 children with vesicoureteral reflux involving 1382 refluxing renal units were evaluated. Among these, 235 renal units demonstrated Grade 1 vesicoureteral reflux. Patients with Grade 1 VUR in duplex collecting systems and those with bilateral reflux in whom the contralateral renal unit demonstrated Grade ≥2 reflux were excluded, resulting in a final cohort of 132 children with 154 Grade 1 refluxing renal units.

Following approval from the Institutional Ethics Committee (Approval No: KAEK 2021/14-232), a retrospective cohort study was conducted at a tertiary pediatric urology center. Medical records of children diagnosed with Grade 1 vesicoureteral reflux (VUR) between January 2008 and January 2025 were reviewed. During the study period, 980 children with vesicoureteral reflux involving 1382 refluxing renal units were evaluated at our institution. Among these, 235 renal units demonstrated Grade 1 VUR. Patient selection was performed at the renal-unit level because eligibility depended on the anatomical characteristics of individual refluxing renal units rather than on patients alone. Patients with Grade 1 VUR in duplex collecting systems and those with bilateral reflux in whom the contralateral renal unit demonstrated Grade ≥ 2 VUR were excluded to obtain a homogeneous cohort in which all refluxing renal units were classified as Grade 1 VUR without contralateral higher-grade reflux. Patients with incomplete follow-up data or unavailable baseline imaging were also excluded. Patients younger than 18 years with unilateral or bilateral Grade 1 VUR confirmed by voiding cystourethrography (VCUG) were eligible for inclusion. Grade 1 VUR was defined according to the International Reflux Study Classification as reflux into a non-dilated ureter without pelvicalyceal involvement. The final study cohort consisted of 132 children with 154 Grade 1 refluxing renal units. The process of cohort identification and patient selection is summarized in Figure 1.

Patients with associated urinary tract abnormalities, voiding dysfunction, recurrent urinary tract infection (UTI), stone disease, familial Mediterranean fever (FMF), or other accompanying conditions were not excluded, as one of the primary aims of the study was to evaluate the clinical significance of associated pathology in children with Grade 1 VUR.

### 2.2. Data Collection

Demographic, clinical, laboratory, imaging, treatment, and follow-up data were collected from institutional electronic medical records.

The following variables were recorded: age at diagnosis, sex, laterality of reflux, number of refluxing renal units, duration of follow-up, history of recurrent UTI, principal associated clinical phenotype, renal ultrasonography findings, DMSA scintigraphy findings, serum creatinine levels, medical and surgical interventions, breakthrough UTI during follow-up, proteinuria during follow-up, and new renal scar formation during follow-up.

Patients were categorized according to the principal clinical phenotype, defined as the dominant clinical presentation or associated condition that represented the predominant clinical context prompting diagnostic evaluation and guiding subsequent management. This classification was intended to describe the clinical context in which Grade 1 VUR was identified rather than to imply a causal relationship between the phenotype and reflux. Accordingly, patients were classified into five predefined categories: (1) voiding dysfunction, (2) recurrent UTI without another identifiable underlying pathology, (3) renal anomalies, (4) stone disease, and (5) familial Mediterranean fever (FMF). When multiple conditions coexisted, each patient was assigned to a single principal clinical phenotype based on the dominant clinical feature considered most relevant to the patient’s presentation and subsequent management. The initial phenotype classification was performed by the primary investigator through detailed review of the complete clinical record according to the predefined study definitions. Cases in which classification was uncertain were subsequently reviewed and discussed with the senior investigators until consensus was reached. Voiding dysfunction was defined as clinically diagnosed lower urinary tract dysfunction and included children with bladder–bowel dysfunction (BBD). Constipation was considered part of the voiding dysfunction phenotype when present as a component of BBD rather than as an isolated condition. The recurrent UTI phenotype comprised children with documented recurrent culture-confirmed urinary tract infections, defined as ≥2 febrile UTIs or ≥3 UTIs in total, when recurrent urinary tract infection represented the dominant clinical presentation prompting evaluation and management. The stone disease phenotype included patients in whom urinary stone disease represented the principal diagnosis prompting clinical evaluation and management. Patients classified within the familial Mediterranean fever phenotype were clinically suspected and genetically confirmed.

### 2.3. Clinical Management and Follow-Up

Patients were managed according to contemporary institutional practice and individualized clinical assessment. Continuous antibiotic prophylaxis was generally preferred for children with recurrent urinary tract infection, voiding dysfunction, or other clinical features considered to increase the risk of recurrent infection, whereas endoscopic intervention was reserved for selected patients with persistent clinical indications despite conservative management. Management decisions were based primarily on the overall clinical phenotype rather than reflux grade alone and included evaluation and treatment of associated clinical phenotypes such as voiding dysfunction, recurrent UTI, constipation, renal anomalies, and urinary stone disease when present. Continuous antibiotic prophylaxis, endoscopic treatment, or additional interventions were performed when clinically indicated. Accordingly, treatment allocation reflected the individual clinical characteristics of each patient and was not intended for comparative evaluation of treatment effectiveness.

After clinical stabilization, routine repeat VCUG was not performed. Follow-up consisted of clinical assessment, urinalysis, urine culture when indicated, renal ultrasonography, and DMSA scintigraphy in selected patients according to clinical requirements. Follow-up DMSA scintigraphy was performed selectively in patients with breakthrough UTI and/or pre-existing renal cortical abnormalities. All patients undergoing follow-up DMSA had an evaluable baseline DMSA examination, allowing paired assessment of interval renal scar formation.

### 2.4. Outcome Definitions

The primary infectious outcome was breakthrough urinary tract infection (UTI). The primary renal outcomes were proteinuria and new renal scar formation. For the purposes of this study, breakthrough UTI was operationally defined as a culture-confirmed symptomatic urinary tract infection occurring after initiation of the planned management strategy, including continuous antibiotic prophylaxis, endoscopic treatment, or expectant management, as appropriate. This definition was applied uniformly regardless of the initial management approach. Proteinuria was defined as persistent proteinuria identified during routine clinical surveillance and confirmed by 24 h urine protein quantification. All patients meeting this definition underwent evaluation by a pediatric nephrologist. Transient isolated proteinuria was not considered an outcome. New renal scar formation was defined as a newly detected cortical defect or scar on follow-up DMSA scintigraphy that was absent on baseline imaging.

A secondary renal adverse outcome was defined as persistent proteinuria and/or newly detected renal scarring. Because follow-up DMSA imaging was performed selectively based on clinical indications, this composite outcome should be regarded as an exploratory endpoint with incomplete ascertainment for incident renal scarring. An exploratory overall adverse outcome was defined as the occurrence of breakthrough UTI or renal adverse outcome during follow-up.

### 2.5. Statistical Analysis

Statistical analyses were performed using SPSS version 27 (IBM Corp., Armonk, NY, USA).

Continuous variables were assessed for normality using the Shapiro–Wilk test. Normally distributed variables are presented as mean ± standard deviation (SD), whereas non-normally distributed variables are reported as median and interquartile range (IQR). Categorical variables are expressed as frequencies and percentages. Patient-level analyses were performed for all clinical outcomes. Renal-unit-level analyses were used only for descriptive variables, including reflux laterality and the distribution of refluxing renal units. Accordingly, new renal scar formation was analyzed as a patient-level outcome, irrespective of whether unilateral or bilateral Grade 1 VUR was present. Comparisons between groups were performed using the chi-square test or Fisher’s exact test for categorical variables and Student’s *t*-test or Mann–Whitney U test for continuous variables, as appropriate. Factors associated with breakthrough UTI and renal adverse outcomes were explored using univariable analyses. These analyses were exploratory and intended to identify unadjusted associations rather than independent prognostic factors. Because of the limited number of renal adverse outcome events, multivariable regression analyses were not performed to avoid model overfitting. All statistical tests were two-sided, and a *p* value < 0.05 was considered statistically significant.

## 3. Results

### 3.1. Patient Characteristics

A total of 132 children with Grade 1 vesicoureteral reflux (VUR) involving 154 refluxing renal units were included in the study. Ninety patients (68.2%) were female, and 42 (31.8%) were male. Bilateral reflux was present in 22 patients (16.7%), whereas 110 (83.3%) had unilateral reflux. The median age at diagnosis was 6 years (IQR 4–9.25). Median follow-up duration was 8 years (IQR 5–9), with a mean follow-up of 7.13 ± 2.58 years. Baseline DMSA scintigraphy was available in 102 patients (77.3%). One examination was unevaluable for classification as normal or abnormal and was therefore excluded from analyses evaluating baseline DMSA status, resulting in 101 evaluable baseline DMSA examinations. Thirty patients (22.7%) did not undergo baseline DMSA evaluation. Follow-up DMSA scintigraphy was performed in 70 patients because of breakthrough UTI and/or pre-existing renal cortical abnormalities. These patients constituted the paired evaluable DMSA subgroup for assessment of new renal scar formation. Accordingly, the frequency of newly detected renal scarring should not be interpreted as an incidence estimate for the entire cohort. Baseline demographic and clinical characteristics are summarized in Table 1.

### 3.2. Principal Associated Clinical Phenotype

A principal associated clinical phenotype was identified in all patients. Voiding dysfunction represented the most common underlying condition and was present in 51 patients (38.6%), followed by recurrent urinary tract infection in 37 (28.0%) and renal anomalies in 33 (25.0%). Stone disease and familial Mediterranean fever were identified in 8 (6.1%) and 3 (2.3%) patients, respectively (Table 2). Among the 33 patients classified within the renal anomalies phenotype, the most frequent abnormalities were renal hypoplasia (*n* = 14), ureteropelvic junction obstruction (*n* = 6), renal agenesis (*n* = 3), duplicated collecting system (*n* = 3), multicystic dysplastic kidney (*n* = 3), horseshoe kidney (*n* = 2), and ectopic kidney (*n* = 2).

### 3.3. Long-Term Outcomes

During follow-up, breakthrough urinary tract infection occurred in 46 patients (34.8%). Among the 46 breakthrough UTIs, 38 (82.6%) occurred during continuous antibiotic prophylaxis, 7 (15.2%) occurred following endoscopic treatment (STING), and 1 (2.2%) occurred during expectant management. Proteinuria developed in 6 patients (4.5%), while among the 70 patients with paired evaluable baseline and follow-up DMSA examinations, new renal scar formation was identified in 7 of the 70 selectively imaged patients with paired baseline and follow-up DMSA examinations (10.0%). Overall, 10 patients (7.6%) developed a renal adverse outcome, including three with proteinuria alone, four with new renal scar formation alone, and three with both outcomes. This composite outcome should be interpreted in the context of selective follow-up DMSA imaging, as incident renal scarring could only be assessed in patients who underwent paired DMSA examinations. An exploratory overall adverse outcome, defined as breakthrough UTI or renal adverse outcome, occurred in 54 patients (40.9%) (Table 3).

### 3.4. Management

Continuous antibiotic prophylaxis was the initial management strategy in 106 patients (80.3%), whereas 14 patients (10.6%) were managed with clinical follow-up alone. Primary endoscopic treatment was performed in 11 patients (8.3%), and one patient (0.8%) underwent ureteroneocystostomy. During follow-up, four additional patients subsequently underwent endoscopic treatment because of persistent clinical indications. Among the initial management groups, breakthrough UTI occurred in 38 of 106 children (35.8%) receiving continuous antibiotic prophylaxis, 7 of 11 children (63.6%) who underwent primary endoscopic treatment, and 1 of 14 children (7.1%) managed expectantly. These data describe the management strategy in place at the time breakthrough UTI occurred and should not be interpreted as comparative treatment effectiveness. Treatment allocation was individualized according to each patient’s clinical characteristics, resulting in substantial confounding by indication and marked differences in group size.

### 3.5. Factors Associated with Renal Adverse Outcomes

Patients were stratified according to the presence of renal adverse outcomes, defined as proteinuria and/or new renal scar formation. Ten patients developed renal adverse outcomes during follow-up.

Patients with renal adverse outcomes were significantly older at diagnosis than those without renal adverse outcomes [13.0 years (IQR 9.8–14.5) vs. 6.0 years (IQR 3.6–9.0), *p* < 0.001]. Among patients with available DMSA scintigraphy, abnormal baseline DMSA findings, defined as pre-existing cortical defects/scarring and/or reduced differential renal function (<45%), were more frequent in the renal adverse outcome group than in patients without renal adverse outcomes [8/10 (80.0%) vs. 38/91 (41.8%), *p* = 0.040]. No statistically significant association was observed between renal adverse outcomes and sex, reflux laterality, renal anomalies, voiding dysfunction, recurrent UTI phenotype, stone disease, FMF, or breakthrough UTI. Detailed comparisons are presented in Table 4.

### 3.6. Factors Associated with Breakthrough Urinary Tract Infection

Breakthrough UTI occurred in 46 patients during follow-up. Female sex was significantly more frequent among patients with breakthrough UTI than among those without breakthrough infection [38/46 (82.6%) vs. 52/86 (60.5%), *p* = 0.009]. Age at diagnosis, reflux laterality, DMSA abnormality, renal anomalies, voiding dysfunction, recurrent UTI phenotype, stone disease, and FMF were not significantly associated with breakthrough UTI. Detailed comparisons are presented in Table 5.

## 4. Discussion

The present study evaluated the long-term clinical course of 132 children with Grade 1 vesicoureteral reflux (VUR) over a median follow-up period of 8 years. The principal findings were threefold. First, clinically relevant infectious and renal outcomes, including breakthrough urinary tract infection (UTI), proteinuria, and new renal scar formation, occurred within this tertiary referral cohort of children with Grade 1 VUR. Second, each child could be assigned to a principal clinical phenotype reflecting the dominant clinical context contributing to presentation and management. Third, renal adverse outcomes were associated with baseline renal parenchymal abnormalities on DMSA scintigraphy and older age at diagnosis in this cohort. However, because these findings derive from exploratory univariable analyses based on a limited number of events, they should not be interpreted as evidence that older age represents an independent biological risk factor. Rather, older age at diagnosis may reflect characteristics of this selected referral population or accumulated disease burden prior to diagnosis. Together, these findings suggest that, in selected referral populations, the clinical significance of Grade 1 VUR may extend beyond its anatomical classification.

Although VUR has been extensively investigated for decades, Grade 1 VUR remains relatively underrepresented as a distinct clinical entity in the literature. Historically, landmark surgical and medical management trials focused predominantly on moderate- and high-grade reflux, whereas more contemporary studies frequently combined Grade 1 VUR with other low-grade categories [7,8,9,10,11,12,13,14]. Consequently, long-term outcome data specifically addressing Grade 1 VUR as an independent clinical entity remain limited. Direct comparison with previous studies is therefore difficult because available evidence largely reflects mixed low-grade reflux populations rather than isolated Grade 1 disease. The RIVUR trial, for example, included children with Grades I–IV reflux, but Grade I represented only a small proportion of the study population [11]. Thus, many of the assumptions regarding the benign nature of Grade 1 VUR have been extrapolated from studies in which these patients constituted only a minority. The present study helps address this gap by examining a relatively large cohort composed exclusively of children with Grade 1 VUR and long-term follow-up.

The most striking observation of the present study was not the presence of Grade 1 VUR itself, but the remarkable heterogeneity of the patients carrying this diagnosis. Each child was classified into a principal associated clinical phenotype based on the dominant associated clinical phenotype contributing to presentation and management. Nearly two-thirds of the cohort demonstrated either voiding dysfunction or a recurrent UTI phenotype, while one quarter had an underlying renal anomaly. These findings suggest that Grade 1 VUR rarely occurred as an isolated radiological finding in our population and instead appeared as part of a broader clinical context. Importantly, reflux grade remained constant across all patients, whereas patient phenotype varied considerably. Within this grade-restricted cohort, substantial heterogeneity in associated clinical features and long-term outcomes was observed. Because the reflux grade was uniform across all patients, the relative prognostic contributions of reflux grade and associated clinical conditions could not be compared.

This concept is further supported by the DMSA findings observed in our study. Importantly, the baseline DMSA abnormalities evaluated in the present study represented pre-existing renal parenchymal abnormalities identified at diagnosis and should be distinguished from incident renal scarring detected during follow-up. Among patients with available scintigraphic evaluation, abnormal baseline DMSA findings were significantly associated with subsequent renal adverse outcomes; 80% of patients who developed renal adverse outcomes had abnormal baseline DMSA findings compared with 41.8% of those without renal adverse outcomes. In contrast, reflux grade was identical in all patients and therefore could not account for differences in outcome. From a nephrological perspective, DMSA abnormalities represent evidence of pre-existing renal parenchymal involvement rather than merely an anatomical urinary tract abnormality [17,18,19]. Previous studies have consistently demonstrated that renal cortical defects and scarring detected on DMSA scintigraphy are among the strongest predictors of long-term renal morbidity in children with VUR [15,20]. Although this association should be interpreted cautiously because DMSA imaging was performed according to clinical indications rather than a standardized protocol, our findings nevertheless suggest that baseline renal parenchymal abnormalities may provide clinically relevant prognostic information within this cohort beyond reflux grade alone. Furthermore, because follow-up DMSA was selectively performed in patients considered at increased renal or infectious risk, the observed frequency of newly detected renal scarring should not be interpreted as an incidence estimate for all children with Grade 1 VUR.

Taken together, these observations support a broader conceptual view of Grade 1 VUR. Within a tertiary referral population, Grade 1 VUR may be encountered in association with clinically relevant urinary or systemic pathology rather than as an isolated benign anatomical finding. In this setting, Grade 1 VUR may be better interpreted as one component of a broader clinical phenotype rather than an isolated anatomical finding. Consequently, the diagnosis of Grade 1 VUR should be interpreted within the broader clinical context of the individual patient, with careful assessment of accompanying conditions, particularly voiding dysfunction, recurrent infection, renal anomalies, and baseline renal parenchymal abnormalities [1,2]. Importantly, our findings do not imply that all patients with Grade 1 VUR require aggressive intervention or surgical treatment; rather, they support individualized management directed toward the accompanying pathology and overall clinical risk profile [2]. In our cohort, most children received continuous antibiotic prophylaxis as part of this individualized management strategy, reflecting contemporary phenotype-based clinical practice rather than treatment directed solely by reflux grade [2].

Another important strength of the present study is its prolonged duration of follow-up. Many previous investigations of low-grade reflux have focused primarily on spontaneous resolution or short-term outcomes [18,21]. In contrast, our cohort was followed for a median of 8 years, allowing evaluation of clinically meaningful renal endpoints such as proteinuria and new renal scar formation, outcomes that may be more clinically relevant to long-term renal prognosis than radiological persistence or resolution of reflux alone [17,20].

The present study has several limitations that should be considered when interpreting the findings. First, this was a retrospective study conducted at a tertiary pediatric urology referral center, resulting in a clinically enriched population that is unlikely to represent the full spectrum of children with Grade 1 vesicoureteral reflux encountered in primary or secondary care. Consequently, the frequency of associated clinical phenotypes and adverse outcomes observed in this cohort should not be generalized to all children with Grade 1 VUR. In addition, the absence of children with isolated Grade 1 VUR, comparison groups without VUR, or other reflux grades precluded evaluation of the independent contribution of reflux relative to the associated clinical phenotypes.

Second, several study variables were collected as part of routine clinical care rather than according to a standardized research protocol. Phenotype assignment was performed retrospectively from the complete clinical records using predefined study definitions, with uncertain cases resolved by investigator consensus. Although this approach reflected routine clinical decision-making, formal assessment of interobserver agreement was not performed. Likewise, follow-up DMSA scintigraphy was obtained selectively in patients with clinical indications, introducing ascertainment and surveillance bias. Consequently, the reported frequency of newly developed renal scarring applies only to patients with paired evaluable DMSA examinations. Similarly, urine screening was performed during routine clinical surveillance rather than according to a standardized schedule, and detailed information regarding orthostatic proteinuria, treatment adherence, febrile status, recurrence timing, and exact timing of individual outcome events was not consistently available.

Finally, the relatively small number of renal adverse outcome events limited the statistical analyses to exploratory univariable comparisons and precluded reliable multivariable modeling. Therefore, the reported associations should not be interpreted as independent prognostic factors but rather as hypothesis-generating observations that warrant confirmation in prospective multicenter studies with standardized longitudinal follow-up and appropriate comparison groups. Nevertheless, the study has important strengths, including one of the largest cohorts composed exclusively of children with Grade 1 VUR, prolonged follow-up, systematic characterization of principal clinical phenotypes, and detailed assessment of clinically relevant infectious and renal outcomes.

Future prospective multicenter studies focusing specifically on Grade 1 VUR are needed to validate these findings, identify the phenotypic characteristics associated with long-term renal outcomes, and develop phenotype-based risk stratification models that move beyond reflux grade alone using appropriate comparison groups.

## 5. Conclusions

In this tertiary referral cohort, children with Grade 1 vesicoureteral reflux demonstrated considerable clinical heterogeneity and experienced clinically relevant infectious and renal outcomes during long-term follow-up. The findings highlight the clinical heterogeneity among children with Grade 1 VUR and suggest that this diagnosis should be interpreted within the broader clinical context, including the principal clinical phenotype and baseline renal parenchymal status. Because of the retrospective design and absence of comparison groups, these findings should be regarded as descriptive and hypothesis-generating. Prospective comparative studies are required to determine whether phenotype-based risk stratification and management improve long-term clinical outcomes.

## Figures and Tables

**Table 1:** Baseline characteristics of the study cohort.

Variable	Value
Number of patients	132
Number of refluxing renal units	154
Female sex, *n* (%)	90 (68.2)
Male sex, *n* (%)	42 (31.8)
Bilateral reflux, *n* (%)	22 (16.7)
Unilateral reflux, *n* (%)	110 (83.3)
Age at diagnosis, median (IQR)	6 (4–9.25) years
Follow-up duration, mean ± SD	7.13 ± 2.58 years
Follow-up duration, median (IQR)	8 (5–9) years
Baseline DMSA performed, *n* (%)	102 (77.3)
Baseline DMSA evaluable for classification, *n* (%)	101 (76.5)
DMSA unavailable, *n* (%)	30 (22.7)

**Table 2:** Principal associated clinical phenotypes of the study cohort.

Associated Clinical Phenotype	*n* (%)
Voiding dysfunction	51 (38.6)
Recurrent UTI	37 (28.0)
Renal anomalies	33 (25.0)
Stone disease	8 (6.1)
Familial Mediterranean fever	3 (2.3)
Total	132 (100)

**Table 3:** Long-term infectious and renal outcomes.

Outcome	*n* (%)
Infectious outcome	
Breakthrough urinary tract infection	46 (34.8)
Renal outcomes	
Proteinuria	6 (4.5)
New renal scar formation ^†^	7/70 (10.0)
Renal adverse outcome *	10 (7.6)
Exploratory overall outcome	
Overall adverse outcome ^‡^	54 (40.9)

^†^ New renal scar formation was assessed only among patients with paired evaluable baseline and follow-up DMSA examinations. Follow-up DMSA was performed selectively according to clinical indications. * Renal adverse outcome was defined as the occurrence of proteinuria (*n* = 3) and/or new renal scar formation (*n* = 4) or both (*n* = 3) during follow-up. ^‡^ Overall adverse outcome was defined as the occurrence of breakthrough urinary tract infection and/or renal adverse outcome during follow-up.

**Table 4:** Factors associated with renal adverse outcomes. Renal adverse outcome = proteinuria and/or new renal scar formation.

Variable	No Renal Adverse Outcome *n* = 122	Renal Adverse Outcome *n* = 10	*p* Value
Age at diagnosis, median (IQR), years	6.0 (3.6–9.0)	13.0 (9.8–14.5)	<0.001
Female sex, *n* (%)	85 (69.7)	5 (50.0)	0.199
Bilateral reflux, *n* (%)	19 (15.6)	3 (30.0)	0.369
Abnormal baseline DMSA findings, *n* (%) *	38/91 (41.8)	8/10 (80.0)	0.040
Renal anomalies, *n* (%)	29 (23.8)	4 (40.0)	0.267
Voiding dysfunction, *n* (%)	48 (39.3)	3 (30.0)	0.740
Recurrent UTI phenotype, *n* (%)	35 (28.7)	2 (20.0)	0.725
Stone disease, *n* (%)	7 (5.7)	1 (10.0)	0.477
FMF, *n* (%)	3 (2.5)	0 (0.0)	1.000
Breakthrough UTI, *n* (%)	44 (36.1)	2 (20.0)	0.493

* DMSA percentages were calculated among the 101 patients with evaluable baseline DMSA examinations. One baseline DMSA examination was technically unevaluable for classification as normal or abnormal. Abnormal baseline DMSA findings were defined as cortical defects/scarring and/or differential renal function < 45% in either renal unit.

**Table 5:** Factors associated with breakthrough UTI.

Variable	No Breakthrough UTI *n* = 86	Breakthrough UTI *n* = 46	*p* Value
Age at diagnosis, median (IQR), years	6.0 (3.0–9.8)	8.0 (5.0–9.0)	0.112
Female sex, *n* (%)	52 (60.5)	38 (82.6)	0.009
Bilateral reflux, *n* (%)	12 (14.0)	10 (21.7)	0.253
Abnormal baseline DMSA findings, *n* (%) *	25/59 (42.4)	21/42 (50.0)	0.448
Renal anomalies, *n* (%)	23 (26.7)	10 (21.7)	0.527
Voiding dysfunction, *n* (%)	35 (40.7)	16 (34.8)	0.506
Recurrent UTI phenotype, *n* (%)	22 (25.6)	15 (32.6)	0.392
Stone disease, *n* (%)	5 (5.8)	3 (6.5)	1.000
FMF, *n* (%)	1 (1.2)	2 (4.3)	0.278

* DMSA percentages were calculated among the 101 patients with evaluable baseline DMSA examinations. One baseline DMSA examination was technically unevaluable for classification as normal or abnormal.

## Data Availability

The datasets generated and analyzed during the current study are available from the corresponding author upon reasonable request. The data are not publicly available due to privacy and ethical restrictions.
